# Modeling a geologically complex volcanic watershed for integrated water resources management in Mt. Fuji, Japan

**DOI:** 10.1038/s41597-025-06380-z

**Published:** 2025-12-06

**Authors:** Stephanie L. Musy, Horst Dresmann, Yama Tomonaga, Yuji Sano, Oliver S. Schilling

**Affiliations:** 1 Hydrogeology, Department of Environmental Sciences, University of Basel, Basel, Switzerland; 2 Applied and Environmental Geology, Hydrogeology, Department of Environmental Sciences, University of Basel, Basel, Switzerland; 3 Entracers GmbH, Dübendorf, Switzerland; 4 Center for Advanced Marine Core Research, Kochi University, Kochi, Japan; 5 Eawag, Swiss Federal Institute of Aquatic Science and Technology, Dübendorf, Switzerland

**Keywords:** Hydrology, Hydrology

## Abstract

This dataset provides high-resolution 3D geological and integrated hydrological models of Mt. Fuji watershed in Japan. The watershed’s complex volcanic and tectonic setting, large spatial extent, and limited subsurface data present significant challenges for integrated hydrological modeling. Diverse geological datasets – borehole logs, geological maps, and hydrofacies surfaces – were collected, processed, and used to construct and validate a 3D geological model suitable for integrated hydrological simulations. Building on this, a 3D numerical model for integrated hydrological simulations was constructed. The repository includes 3D hydrofacies surfaces in raster format, numerical mesh files, and input configurations necessary to run simulations with the integrated surface-subsurface hydrological simulator HydroGeoSphere. The preparation of heterogeneous geological data, construction of hydrofacies surfaces, generation of the numerical mesh, and setup of the integrated hydrological model are described in a streamlined, reproducible workflow suited for volcanic contexts and transferable to other geologically complex or data-limited regions. These resources are intended to reduce trial-and-error iterations and support further research in groundwater assessment, model calibration, climate impact studies, and hazard mitigation.

## Background & Summary

In an evolving global economy marked by increasing resource scarcity and rising energy demands, effective resource and environmental management has become highly dependent on advanced modeling techniques^1^. Among these, subsurface geological modeling plays a crucial role in addressing both society’s reliance on subsurface resources and the significant environmental impacts associated with their extraction. Additionally, geological heterogeneities across multiple scales contribute to natural hazards, including earthquakes, volcanic eruptions, floods, and droughts, underscoring the importance of accurate geological modeling. In this context, three-dimensional (3D) geological models have emerged as indispensable tools across a wide range of applications, from deep geological repository management to oil and gas exploration, mining, water resources management, and geological hazard assessment^2^.

3D structural geological models form the foundation for the spatial parameterization of subsurface resource management tools, including groundwater flow models and deep geological repository assessments. Accurate groundwater models are critical for informed decision-making and are increasingly central to shaping current and future water management policies. The most physically robust approach to hydrological modeling is through Integrated Surface–Subsurface Hydrological Models (ISSHMs), which simulate all components of the water cycle in a fully coupled, physically based, and spatiotemporally distributed manner^3–6^. These models support predictive analyses of surface water-groundwater interactions under changing environmental conditions and under explicit consideration of interactions with the atmosphere, the biosphere, and the cryosphere. Through this, ISSHMs enable detailed forecasting of future key hydrological parameters, such as groundwater recharge rates, evapotranspiration, headwater dynamics, and seasonal river flow distribution, while allowing the impacts of land-use and climate change to be assessed^7–10^. Beyond technical applications, ISSHMs serve as powerful communication tools. By offering a clear, accessible representation of processes that are otherwise difficult to visualize, they enhance understanding among non-specialist stakeholders and support well-informed decisions in water and subsurface resource governance^11^. This aligns directly with the United Nations Sustainable Development Goal (SDG) 6, which aims to ensure the availability and sustainable management of water and sanitation for all. As such, ISSHMs are increasingly recognized as essential to the implementation of integrated water resources management strategies^12^ and have been used to support regional- to continental-scale hydrological simulators (e.g., the Japan National Water Cycle Model (Geosphere Environmental Technology Corp., 2025), Canadian Continental Scale Model^13^, Continental scale model of the United States of America^14^, or the Pan-European Terrestrial Hydrological Model^15^).

Volcanic island states are among the regions that are most urgently in need of resilient and sustainable water management strategies. These regions face a unique convergence of challenges: the typical pressures of coastal environments, such as flooding, sea-level rise, and saltwater intrusion^16^, combined with mountainous conditions, including steep hydraulic gradients, complex geology, and rugged topography^6^. Additionally, their tectonic setting subjects them to heightened volcanic and seismic risks^17^. Island states located along the Pacific Ring of Fire–including Japan, Taiwan, the Philippines, and Indonesia–host the majority of the world’s active volcanoes and account for approximately 80% of global seismic activity. In many volcanic island regions, coastal volcanic aquifers serve as the primary freshwater source, with few, if any, viable alternatives due to their geographic isolation. This dependency is further compounded by high population densities in coastal megacities (e.g., Tokyo, Yokohama, Osaka, Nagoya, Taipei, Jakarta, and Manila), where pressure on volcanic aquifers bordering the coast is particularly acute. While in Japan, strict groundwater abstraction regulations–implemented to mitigate land subsidence^18^–have reduced current extraction in some coastal cities, other parts of the Asia-Pacific region still experience high reliance on coastal volcanic aquifers^19,20^. Given these dynamics, hydrogeological modeling not only plays a critical role in the sustainable management of subsurface resources such as groundwater and geothermal energy, but also in disaster preparedness (e.g., earthquakes, volcanic unrest, tsunamis). In this context, ISSHMs offer the most comprehensive framework for evaluating both water-related potentials and risks^21–25^. They enable simultaneous analysis of water quantity and quality, above and below the surface, and are critical tools for planning and resilience building in some of the world’s most vulnerable regions^26^.

In response to these challenges, we present an openly accessible dataset developed to support the construction of 3D geological models for use in integrated hydrological modeling of volcanic aquifer systems. This dataset was produced for Mt. Fuji watershed in Japan, a large, geologically complex and data-scarce environment, using a streamlined step-by-step workflow for the integration of diverse geological datasets, including stratigraphic layers and borehole profiles. The workflow describes the definition of hydrogeologically meaningful hydrofacies, the computation and refinement of these surfaces for ISSHM construction, numerical mesh generation, and the export of these layers into ISSHM-compatible formats. Subsequently, the procedure to build an ISSHM, which supports hind- and nowcasting as well as projection of climate change impacts, is provided. This approach reduces the trial-and-error iterations typically required to develop a functional integrated hydrological model and thus, it improves environmental resource management and disaster preparedness in countries and cities facing the most serious climate change challenges.

The datasets generated in this study are freely available for use, modification, and visualization. They are intended to improve accessibility for a wide range of users, including researchers in volcanic hydrogeology, governmental agencies managing water resources and disaster preparedness, and technical practitioners involved in environmental planning, subsurface exploration, or water supply management. By openly sharing both the data and the methods used to produce them, this work supports transparency, reusability, and broader adoption of high-resolution hydrological modeling in complex geological settings.

## Study area: Mt. Fuji catchment, Japan

**Fig. 1:**
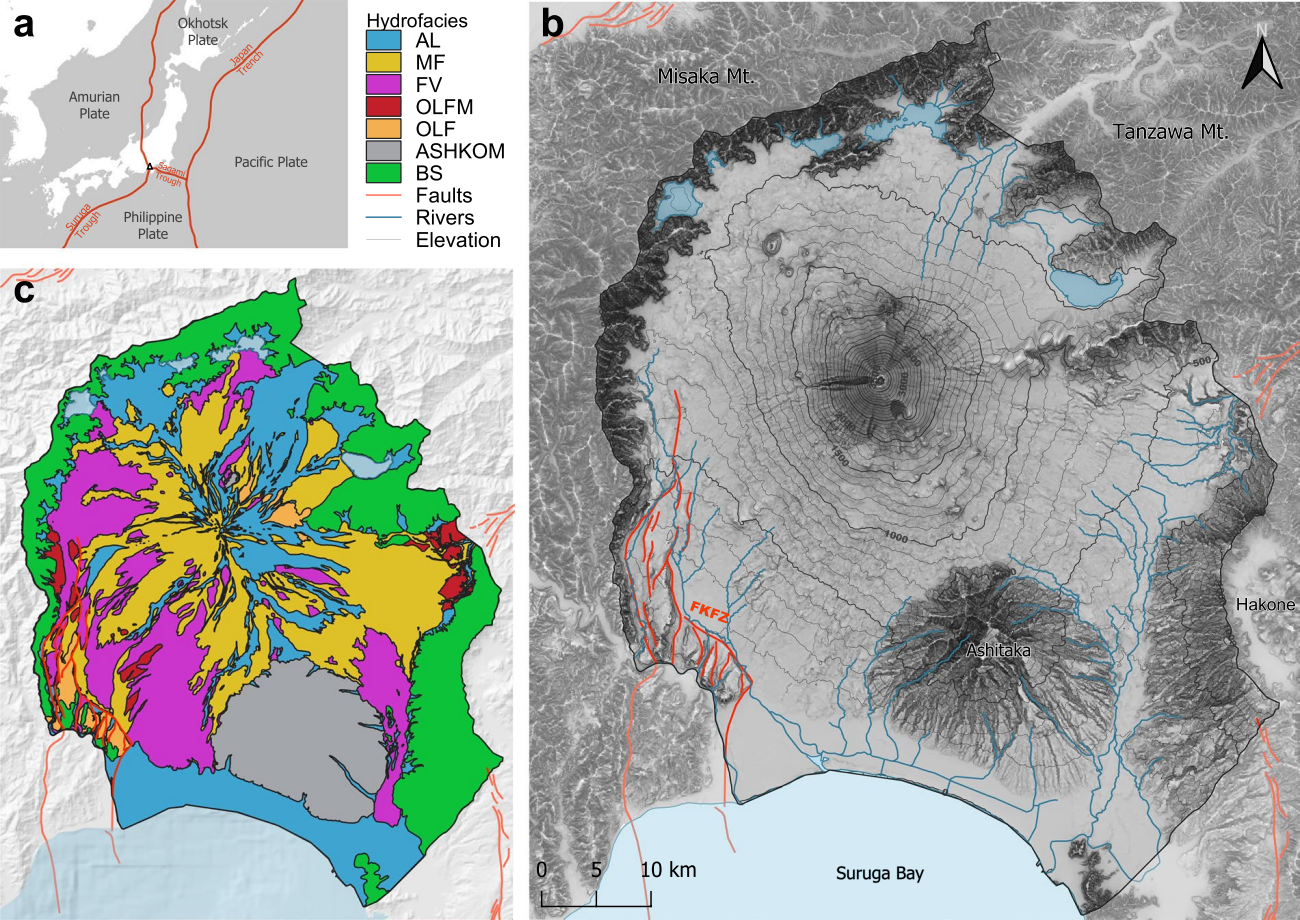
(**a**) Tectonic context of Mt. Fuji, located on the triple-trench junction between the Pacific, Philippine, Okhotsk and Amur plates; (**b**) Map of Mt. Fuji catchment, its surrounding mountains, active tectonic faults (red), hydrological network (simplified rivers and Yamanaka, Kawaguchi, Sai, Shoji, and Motosu lakes, from east to west), and elevation isolines. (**c**) Geological Map of Fuji volcano with layers grouped into the seven hydrofacies relevant for this study (see Table 1 for the hydrofacies description). Composite map sources: hillshade relief map: Red Relief Image Map^79,132^; active tectonic fault locations^64^; digital elevation model^83^; Geological map^59^.

The Japanese archipelago, part of the Pacific Ring of Fire, lies at the convergence of four tectonic plates (Fig. 1a), making it one of the most seismically active regions on Earth. The country, which consists of 14,125 islands and is home to over 100 active volcanoes, has a history marked by major seismic events, including the devastating Kobe earthquake (1995) and the more recent Tohoku-Oki earthquake (2011), which triggered the tsunami that led to the Fukushima disaster^27,28^. Mt. Fuji—Japan’s tallest and most iconic peak—is an active, albeit dormant, volcano that hosts one of Japan’s most crucial watersheds, supplying freshwater to millions for domestic, agricultural, and industrial use^29^. Mt. Fuji’s groundwater is one of the most vanadium-rich on Earth, a characteristic that has drawn both local and global attention in the form of an ever-growing mineral water bottling industry^30–32^. However, in recent years, Fuji’s water resources have come under increasing pressure due to pollution from these industrial and agricultural activities, as well as from over-extraction, which is primarily driven by the rising domestic and international tourism^33^.

Unfortunately, despite decades of research on Mt. Fuji’s geology and hydrogeology^30,32–43^, critical knowledge gaps regarding groundwater origins and flow paths, residence times, recharge and discharge processes, and catchment-scale hydrodynamics have persisted for a long time. Although to date several regional conceptual and numerical sub-models have been constructed and an integrated national hydrological model that covers the entirety of Japan has been built (i.e., the Japan National Land Water Cycle Model), no comprehensive, catchment-wide conceptual - and even less so - numerical integrated hydrological model has been developed. These knowledge gaps have become increasingly pressing due to the compounding dangers of water pollution, over-extraction, climate change, and seismic activity. Recent events—such as earthquake-induced groundwater flooding (e.g., after the 2011 Shizuoka Earthquake, which had an epicenter was located near Mt. Fuji’s magma chamber^33,44^), declining lake water levels^44,45^, and seawater intrusion in Suruga Bay^46^— further underscore the need for advanced, site-specific modeling^33^.

Given these challenges, an ISSHM is crucial for the sustainable and conjunctive management of groundwater and surface water resources and disaster preparedness in the Mt. Fuji region. As such, Mt. Fuji serves as an ideal case for demonstrating a workflow for complex volcanic island watershed geological and integrated hydrological model development.

### Geography

At 3,776 m ASL, Mt. Fuji is Japan’s highest peak, located about 100 km southwest of Tokyo^47,48^ (Fig. 1). The upper slopes of the mountain exhibit alpine climatic conditions, characterized by extended dry periods (Köppen climate classification: Dfc/ET), while the lower elevations experience a humid temperate climate, with abundant water resources (Köppen climate classification: Cfa)^49^. Precipitation patterns vary significantly across the mountain, with the wettest slopes (south and east) receiving 2,700–3,000 mm yr^−^¹, and the northern slopes^50^ receiving 1,500–2,000 mm yr^−^¹. To the north, Mt. Fuji is surrounded by the Fuji Five Lakes (Kawaguchi, Yamanaka, Sai, Motosu, and Shōji), which were formed when lava flows dammed pre-existing river valleys^51,52^. Groundwater contribution to the lakes is likely limited, and their water level is mainly controlled by a balance between rain and evaporation^53^. Notably, these lakes have no natural outflows, except for Lake Yamanaka, which is drained by the Katsura River towards the northeast^47^. It is estimated that approximately 75% of the total precipitation infiltrates into the subsurface, recharging the regional groundwater system^50^. This groundwater emerges as thousands of springs at the base of Mt. Fuji^32,54,55^, supplying high-quality, mineralized water to local communities. The watershed is drained by three major rivers, all of which originate from groundwater resurgences: the Ayuzawa River on the eastern, the Fuji River on the southwestern, and the Kise-Kano Rivers on the southeastern flank. Furthermore, submarine groundwater discharge (SGD) has been documented in Suruga Bay^46,56^.

### Tectonic context

Mt. Fuji is situated directly on top of the only known continental trench-trench-trench triple junction on Earth, where the Amurian (West Japan), Okhotsk (East Japan), and the Philippine Sea Plates (Izu Peninsula) converge. The plate boundaries between the Philippine Sea and the Amurian Plates and between the Philippine Sea and the Okhotsk Plates are defined by the Suruga Trough and the Sagami Trough, respectively^48^ (Fig. 1a).

While most Japanese volcanoes are predominantly composed of andesitic lava, Mt. Fuji is the largest basaltic polygenetic volcano in Japan^34^, with an estimated volume of 400–500 km^3^. This distinction is linked to the unique position of Mt. Fuji’s magma chamber, which lies within a gap between the two subducting segments of the Philippine Sea Plate beneath the volcano^57^. This unusual structural setting contributes to Mt. Fuji’s high volcanic variability, influencing eruption frequency, volume, location, and type^58–61^. Seismic activity beneath Mt. Fuji is also notable. Low-frequency earthquakes occur at depths of 15–20 km^62^, likely associated with the feeding processes of a stable magma reservoir that continually supplies the volcano. This reservoir is estimated to be located at approximately 20 km depth^34,57,62,63^.

The Fujikawa-Kako Fault Zone (FKFZ) is a major tectonic feature in the region, forming the northern extension of the Sagami Trough and crossing the southwestern foothills of Mt. Fuji (Fig. 1b). It is recognized as one of the most active fault zones in Japan^64^. The FKFZ consists of multiple fault segments with a general north-south orientation and a westward dip^65^ of 80–85°. The Iriyamase Fault, the primary branch of the FKFZ, is characterized by reverse faulting, with westward uplift and a long-term average slip rate^66^ of 10 m kyr^−1^. The last major rupture event in this fault system occurred in the 7th or 8th century BC^48,59^. Evidence suggests that older Fuji lava deposits have been uplifted by as much as 100 meters on the western side of the fault. Geophysical studies have identified a highly conductive anomaly beneath the FKFZ, hypothesized to represent fluids trapped within the deep fault zone. While its exact mechanism remains uncertain, this anomaly is suspected to influence subduction-zone seismicity in the region^57^.

### Formation history and geological context

The geology surrounding Mt. Fuji, including the Misaka and Tanzawa Mountains, consists primarily of Miocene accretionary complexes, such as the Tanzawa Group and Nishiyashiro Group to the west, north, and east. These units are overlain by Pliocene through-filling sediments composed of sandstone, mudstone, and conglomerate (Table 1)^44,67–69^. Mt. Fuji is also surrounded by Quaternary volcanic ejecta, originating from Hakone Volcano to the southeast and Ashitaka Volcano to the south^70^ (Fig. 1b). Prior to the formation of Mt. Fuji, the region was dominated by the Pre-Komitake (270,000–160,000 yrs BC) and Komitake (100,000 yrs BC) volcanoes, which form the northern and northeastern Quaternary basement of the current Mt. Fuji edifice. These early volcanoes are distinguishable by topography and petrography, as they primarily produced andesitic and basaltic-andesitic lava^61^. The formation of Mt. Fuji itself began more than 100,000 years ago, during the Pleistocene Epoch^61^. Its volcanic activity is divided into several stages, based on (i) structural geology differences between erupted materials (e.g., older deposits were significantly displaced by the activity of the FKFZ^65^; (ii) Stratigraphic order, distinguishing key eruption sequences; and (iii) Types of volcanic activity, including variations between pyroclastic eruptions and effusive lava flows. Traditionally, Mt. Fuji’s history was divided into two broad stages: Ko-Fuji (Old Fuji) and Shin-Fuji (Young Fuji)^71,72^. However, many recent studies have proposed more detailed classifications^48,59^. The Hoshiyama Stage, which lasted from approximately 100,000 to 15,000 years BC, corresponds to what was formerly referred to as Ko-Fuji. This period was dominated by explosive eruptions, which were frequently accompanied by widespread mudflows and extensive ash deposition. Towards the end of this stage, around 17,000 BC, the activity transitioned into a phase characterized by massive lava effusions and increased mudflows, setting the foundation for the subsequent construction of the modern volcanic edifice^60,61,72^. Following the Hoshiyama Stage, the Fujinomiya Stage began around 15,000 BC and lasted until approximately 6,000 BC. This period was marked by the emplacement of extensive basaltic lava flows, which played a critical role in shaping the contemporary structure of Mt. Fuji. The accumulation of these flows gradually formed a more stable volcanic cone^58,59^. The most recent stage of volcanic activity is known as the Subashiri Stage. During this period, volcanic activity has fluctuated between high- and low-intensity phases, leading to a more complex classification of this stage into four sub-periods (Table 1). The earliest phase, from 6,000 to 3,600 years BC, was characterized by low volcanic activity and followed by an active phase lasting until 1,500 years BC. The subsequent period, spanning from 1,500 to 300 years BC, continued to witness significant lava flows and pyroclastic activity before giving way to the most recent phase, which extends from approximately 300 years BC to today^66,71^. Two of Mt. Fuji’s most well-documented eruptions occurred during this last phase. The Jogan eruption, which took place between 864 and 866 AD, was one of the earliest directly recorded eruptions, producing extensive lava flows. The most recent eruption, the Hoei eruption of 1707 AD, occurred at three craters on the southeastern flank of the volcano. This eruption, however, did not produce lava flows but resulted in widespread ashfall reached as far as Tokyo, covering vast areas in volcanic debris^73^.

**Table 1:** Volcanic formation history, resulting rock facies, and key hydraulic properties (e.g., hydraulic conductivity, anisotropy, and effective porosity) for the seven hydrofacies as defined in this study.

Period	Epoch	Age	Stratigraphy			Geological features			Hydrogeological properties			
						Volcanic activity	Rock facies		Hydrofacies	Hydraulic conductivity	Anisotropy	Effective porosity
		Ref. ^44^				Refs. ^48,59,70^	R ^44,48,59,61,66,68–72,79,80^			[m s^-1^]	K_V_/K_H_	[%]
Quaternary	Holocene	Today	Shin-Fuji			Alluvium; Topsoil; Volcanic ash		Sedimentary layers: Gravel and sand	**AL**	1 × 10^-4^	1	30
		1,700 AC		Subashiri stage	d	Flank lava extrusion (incl. Hoei and Jogan eruptions)		Young volcanic gravel; Lava flows				
		0 AC			c	Explosive eruptions and collapses of flanks		Debris and thick lava flows; Scoria fall deposits	**MF**	1 × 10^-5^	1	10
		2,000 BC			b	Central cone building		Lava flows intercalated with pyroclastic material (volcanic gravel, sediments)				
		4,000 BC			a	Low volcanic activity						
		6,000 BC		Fujinomiya stage		Continuous voluminous lava extrusions		Lava flows, scoria fallouts and sand (Pyroclastic cones)	**FV**	1 × 10^-3^	1/10	20
	Pleistocene	15,000 BC										
		20,000 BC	Ko-Fuji	Hoshiyama stage		Explosive eruptions followed by major collapses of the edifice		Mudflows, fan deposits	**OLFM**	1 × 10^-6^	1/100	10
		30,000 BC						Massive basaltic lava flows	**OLF**	1 × 10^-4^	1/10	15
								Scoria deposit - Volcanic gravel, sand and silt				
		100,000 BC	Komitake and Pre-Komitake					Basaltic - Andesite lava with low K_2_O and TiO_2_ content	**ASHKOM**	1 × 10^-6^	1/10	15
			Ashitaka Volcano					Loam layer - Basaltic to andesitic lavas and pyroclastic flow deposits				
			Hakone Volcano					Basaltic to andesitic lavas and pyroclastic flow deposits	**BS**	1 × 10^-8^	1	5
			Iwabuchi Volcano					Basaltic to andesitic lavas and volcaniclastic rocks				
Neogene	Plio-cene					Accretionary complexes forming the Misaka and Tanzawa mountains		Sandstone, conglomerate, mudstone and tuff				
	Mio-cene	Fujikawa (SW), Nishiyatsushiro and Tanzawa (N-E) groups										

The hydrogeological properties are based on field observations (borehole investigations, pumping tests) and numerical modeling results from the National Institute for Advanced Industrial Science (AIST)^128^, which focused on the southwest of the Mt. Fuji groundwater system. The indicated age refers to the younger (upper) boundary of each stratigraphic interval.

Today, Mt. Fuji is classified as a dormant volcano, having remained inactive for over three centuries. Despite its long dormancy, it remains capable of future eruptions. Volcanologists now believe the next eruption is overdue, especially after the 2011 M9 earthquake that reactivated seismic activity beneath the mountain^74^. Predicting future volcanic activity remains challenging due to the high variability in eruption intervals and the volume of material released throughout its history^59,60^. In response to these concerns, in March 2025, the Mt. Fuji Volcano Disaster Prevention Council issued its first-ever evacuation plan for the eventuality of an eruption of Mt. Fuji.

### Hydrogeological stratigraphy

Volcanics such as the Quaternary formations of Mt. Fuji are typically highly permeable and form excellent multilayered aquifer systems. The volcanic edifice of Mt. Fuji, for example, is characterized by a complex groundwater flow system, where recharge primarily occurs in the upper regions of the mountain. From there, groundwater percolates downward through the flanks, flowing under confined conditions along the porous matrix and highly permeable clinker zones, which form above and below the dense, massive central cores of basaltic lava flows. The formation of these clinker-like structures is controlled by the thermal gradient during lava solidification: as the lava cools, the outermost layers lose heat more rapidly than the central core, leading to the formation of a fragmented, porous structure at the top and bottom of the flow. In many cases, groundwater emerges at the termini of outcropping lava flows, forming numerous large springs in Mt. Fuji’s foothills.

The geometry and permeability of lava flows vary with elevation, which in turn influences groundwater movement. In the upper part of the mountain, lava flows are typically steeper, narrower, and thinner, with well-developed clinker zones. Precipitation and snowmelt infiltrate these clinker-rich layers and percolate downward, ultimately contributing to deeper groundwater flow. Conversely, on the mid- to lower slopes, the dense central portions of lava flows act as hydraulic barriers, preventing water from infiltrating deeper layers. As a result, precipitation and surface water in these areas accumulate as shallow groundwater, forming what is known as surficial groundwater flow^44^. In addition to lava flow deposits, mudflow deposits play a significant role in the hydrogeological framework. These deposits contain interbedded gravel layers with relatively high permeability but also include impermeable mud layers that significantly reduce overall hydraulic conductivity. Consequently, these mudflow deposits tend to act as hydrogeological barriers, dividing groundwater movement within the volcanic stratigraphy^70^.

Stratovolcanoes, such as Mt. Fuji, are formed by successive eruptions that deposit volcanic material unevenly around the summit. As a result, the volcanic stratigraphy of Mt. Fuji is extremely complex, with approximately 200 distinct volcanic layers (i.e., lithofacies) identified^59,75^. Capturing this degree of heterogeneity in a hydrogeological model is impractical, necessitating a simplification of the lithographic stratigraphy into hydrofacies, which group units with similar hydrogeological properties and deposition histories^76,77^. Several previous studies have proposed region-specific hydrogeological classifications. For example, former studies focused on the northern^44^, eastern^40^, and southwestern^31^ foothills, but a catchment-wide consistent classification has so far been missing. And while the Japan National Water Cycle Model, developed by Geosphere Environment Technology Corp. (GETC) and referred to below simply as the National Hydrological Model (NHM)^78^, does build on catchment-scale hydrofacies for consistent national-scale hydrological simulations, the employed hydrofacies classification and spatial extents are comparably coarse (500 m × 500 m horizontal resolution) and consequently not fully consistent with sub-catchment or local-scale geological structures. It is, however, these sub-catchment structures that are critical for consistent local-scale water resources management.

As a comprehensive, catchment-wide conceptual hydrogeological model of Mt. Fuji has not yet been developed, we present a regionally consistent conceptual model for Mt. Fuji’s most important hydrofacies based on a synthesis of existing geological and hydrogeological studies^44,48,59,61,66,68,70–72,79–81^. Table 1 summarizes the volcanic formation history, resulting rock facies, and key hydraulic properties (e.g., hydraulic conductivity, anisotropy, and effective porosity) of the seven hydrofacies used in this study, while the hydrogeological reasoning behind the definition of each hydrofacies is described below.

The topsoil and alluvium consist of recent volcanic and alluvial sediments, primarily unconsolidated, and are distributed along Suruga Bay, as well as the Fuji, Urui (SW), and Kise (SE) Rivers, and on the eastern and northern slopes of Mt. Fuji. These deposits correspond to the final phase of the Subashiri stage (Subashiri-d), and due to their shallow depth and similar hydraulic properties, they have been grouped into one single hydrofacies (**AL**), forming a surficial, unconfined aquifer. The volcanic mudflow deposits and sediments of the Subashiri-a and -b, as well as parts of Subashiri-c, stages, have been classified as a single hydrogeologic formation (**MF**). This unit is separated from the AL hydrofacies by less-permeable mudflow and sediment deposits from the Subashiri-c stage^44^. The underlying Fujinomiya Stage volcanic ejecta and lava flows (**FV**) hold a significant volume of groundwater. This unit corresponds to what was previously referred to as the “Shin-Fuji” or “Young Fuji” aquifer^40^. Below this, the Old Fuji (“Ko-Fuji”) Mudflow (**OLFM**), a basaltic volcanic mudflow ejected at the end of the Hoshiyama Stage, forms an important aquitard. Mt. Fuji’s largest springs, such as the UNESCO World Heritage-designated Kakitagawa Spring and Shiraito Falls, emerge from the contact between the OLFM and FV formations, supporting the assumption of OLFM acting as a largely impermeable layer^32,44^. However, significant seepage and upwelling across the OLFM have been documented in different locations around Mt. Fuji, particularly around the FKFZ^32,82^. The volcanic ejecta of the earlier Hoshiyama Stage (**OLF**) consist of lava and gravel layers with limited mudflow intercalations, making them another important aquifer for the region. Below these formations, the Komitake and Ashitaka volcanic ejecta (**ASHKOM**) are predominantly andesitic and loamy, characterized by low hydraulic permeability. The hydrogeological basement (**BS**) of the region consists of Lower Pleistocene formations (including the Hakone Volcano and Ashigara groups and the Iwabuchi Volcano) and Neogene formations (such as the Misaka Formation). These units, composed primarily of sandstone, stratified conglomerate, mudstone, and tuff, are highly solidified and act as the regional hydrogeologic base^68^. Water from the basement is typically warmer, and thus locally exploited for onsens (i.e., traditional Japanese thermal mineral baths).

## Methods

### Input data

Table 2 summarizes the input data used in the 3D geological and 3D hydrological model-building workflow described in this study. It compiles information on hydrofacies interfaces, including surface topography, bathymetry, hydrogeological units, fault systems, and overland flow parameters, as well as the URL where the data can be found and their references.

**Table 2:** Type, Name, References, URLs, characteristics, and format for the input data used in the construction and verification of the 3D geological and hydrogeological models; all URLs were last accessed in June 2025.

Type	Name	Reference	URL	Characteristics	Format
Digital Elevation Model (DEM)	ASTER Global Digital Elevation Model (GDEM) V003	NASA/METI/AIST/Japan Spacesystems/U.S./Japan ASTER Science Team^83^	10.5067/ASTER/ASTGTM.003	1 arc-sec (≈30 m) spatial resolution	Raster (.tif)
Ocean bathymetry	General Bathymetric Chart of the Oceans	GEBCO Bathymetric Compilation Group^84^	https://www.gebco.net	15 arc-sec (≈450 m) spatial resolution	Raster (.tif)
Lake bathymetry	Lake and Pond bottom elevations	Geospatial Information Authority^85^	https://www.gsi.go.jp/kankyochiri/lakedatalist.html	10 m spatial resolution	Raster (.tif)
Hydrological network	All Rivers, All Lakes & Topography across all Japan	MLIT^87,88^.	https://nlftp.mlit.go.jp/ksj/index.html	River centrelines and lake surface areas across the Japanese archipelago; 1:25,000 scale	Vector (.shp)
Hydrogeological units interfaces	National-scale hydrological model (NHM): Distribution areas and lower surface contours of hydrogeological units	Geosphere Environment Technology Corp. (GETC)^78^	https://www.getc.co.jp/webmap_en/	designed for 500 m x 500 m horizontally resolved national scale hydrological simulations, Integrated in the National Land Information Platform of Japan	Vector (.shp)
	Geological Map of Fuji Volcano	Takada *et al*.^59^	https://www.gsj.jp/Map/EN/docs/misc_doc/misc_12_2nd.html	1:50,000 spatial scale, second edition	Vector (.shp)
	Topography of Mishima lava flow terminus (FV)	Tsuchi (2017)^40^		1:20,000 map in Mishima City with contour lines	Map (.jpg)
	Upper and lower surface elevation lava and mud layers from Fujinomiya and Hoshiyama stages	Murashita (1982); Ono *et al*.^31,116^		1: 200,000 map in the Fuji City area (SW) with contour lines of OLF, OLFM, FV and MF	Map (.jpg)
	Cross-sections throughout the catchment	Ono *et al*.^68^		1:400,000 map of the whole catchment with cross-section traces	Image (.jpg)
Verification boreholes	Discrete locations of the bottom elevation of the MF, FV and OLFM layers	Ikeda (1989); Uchiyama (2020); Yamamoto^44,80,129^		Borehole georeferenced and depth reported for each layer interface	Vector (.shp)
Faults	Position of the major branches of the Fujikawa-Kako Fault Zone	Active fault database of Japan^64^	https://gbank.gsj.jp/activefault/index	Lines compiled from the 1:200,000 map; General N 10°E direction, 30 km length	Vector (.shp)
Land-use and land-cover	Land-use and land-cover map of Japan	Japan Aerospace Exploration Agency, Earth Observation Research Center, version 23.1.2^92^	https://www.eorc.jaxa.jp/ALOS/en/dataset/lulc_e.htm	Integrates Sentinel-2, Landsat-8, and ALOS-2 (PALSAR-2) satellite data; Overall classification accuracy: 95.53%; spatial resolution: 100 m	Raster (.tif)

#### Digital Elevation Model (DEM)

To define the top of the modeled domain, a sufficiently well-resolved DEM was required. Surface topography was represented using the ASTER Global Digital Elevation Model (GDEM Version 3^83^), which offers a spatial resolution of 1 arc-second (approximately 30 m). For the submarine topography in the Suruga Bay area, bathymetric data from the GEBCO_2024 (General Bathymetric Chart of the Oceans^84^ were merged with the land DEM. In addition, lake bathymetry data provided by the Geospatial Information Authority of Japan^85^ were appended to accurately represent lake bottoms within the study area. A similar but global product, the GLOBathy dataset^86^, is also available and offers comparable bathymetric information.

#### Hydrological network

To accurately represent surface water features in the hydrological cycle, geographic information from the national river and lake maps was used^87,88^. The datasets include river centrelines and lake surface areas across all of the Japanese archipelago, based on a 1:25,000 scale. The coarser, global HydroRIVERS^89^ and HydroLAKES^90^ datasets could be considered as alternative data sources with global coverage where more highly resolved local datasets are unavailable.

The current integrated hydrological model implementation focuses on the natural hydrological system, excluding anthropogenic modifications such as dams, streamflow regulation structures, and municipal irrigation networks. This approach allows for a clearer baseline understanding of natural hydrodynamics within the catchment.

#### Hydrofacies boundary surfaces

As a basis for the construction of the highly resolved, catchment-scale hydrofacies used in this study, existing information from the NHM (500 m x 500 m horizontal resolution^78^ was used. The digitized hydrofacies information as implemented in the NHM was obtained directly from GETC and supplied as shapefiles, containing contour lines of the bottom bounding surface of each hydrofacies as well as polygons defining their horizontal extent. These shapefiles are provided in the data repository associated with this paper^91^.

While the existing hydrofacies product of the NHM includes estimated surfaces of all major hydrofacies of Mt. Fuji catchment, the model was not designed to be consistent at the sub-catchment or local scale, and as a result, some surfaces were absent or discontinuous in certain areas of the catchment, or defined too coarsely to match the detailed geological structures relevant at the sub-catchment scale. To produce hydrologically robust simulations of Mt. Fuji catchment, additional data were thus needed to: (i) complete the near surface hydrostratigraphy across most areas, (ii) complete and fill internal gaps in the existing hydrofacies using current geological information to ensure full spatial coverage, and (iii) improve resolution in geologically complex areas.

The following information sources and input data were thus considered: surface (i.e. outcrop) information from the geological map of Fuji Volcano, hydrogeological layers’ bottom elevation contours from localized studies around the catchment, and borehole logs and geological cross-sections from both geological and hydrogeological studies on Mt. Fuji.

It is important to note that in the case of the Mt. Fuji catchment, a preliminary hydrofacies surface dataset was available and served as the foundation for the workflow presented here. In regions where such datasets are not available, equivalent hydrofacies surfaces can be constructed from geological cross-sections, borehole records, and other stratigraphic information by following the same workflow outlined in this study.

#### Faults

Data for the Fujikawa-Kako Fault Zone were obtained from the Active Fault Database of Japan^64^. Information on faults is essential not only for accurately modeling the geological structure, but also for assigning hydrogeological parameters and understanding flow discontinuities or barriers in hydrological models. Although the FKFZ comprises numerous conjugate faults, for modeling purposes this study simplified the system to two major fault traces. The fault system is about 30 km long and oriented towards the North with a 10° angle towards the East. Given likely variations along the fault zone, both traces were conceptualized as predominantly vertical structures, consistent with previous studies^65,75^.

A significant contrast in aquifer thickness was observed across the FKFZ: aquifers on the eastern side reach depths of up to 300 meters, while those on the western side are generally limited to around 100 meters.

#### Land-use and land-cover

To accurately parameterize overland flow in the integrated hydrological model, land-use and land-cover (LULC) data were used. This information was obtained from the 2022 High-Resolution Land-Use and Land-Cover Map of Japan (v23.12), developed by JAXA’s Earth Observation Research Center (EORC)^92^. This dataset includes 14 land-cover categories, which are later grouped into 5 hydrologically relevant categories.

### Software

#### Geographic information system

A geographic information system (GIS) software was used to pre-process and post-process the model’s input and output files. In other words, it was used to convert data into the formats required by geological and integrated hydrological modeling and visualization tools, as well as to present some of the results. In this study, the free and open-source QGIS platform (QGIS Association; version 3.38.2; https://www.qgis.org) was used. Alternatively, the commercial software ArcGIS Pro (ESRI; https://www.esri.com) could also be used.

#### Geological modeling software

A 3D geological modeling software was used to create detailed representations of subsurface structures by integrating various geological data, and to visualize complex geology and supports analysis and simulations. Here, we chose the commercial software Aspen SKUA (Aspen Technology Inc.; SKUA-GOCAD Paradigm 19; https://www.aspentech.com/en/products/sse/aspen-skua). Alternatively, the free and open-source GemPy software^93^ (https://www.gempy.org) could be used.

#### Numerical mesh generation software

A numerical mesh generation software was required for the generation of a triangular finite element mesh. For the simulation of geologically and hydrologically complex systems such as volcanic island aquifers, and specifically Mt. Fuji watershed, a powerful mesh generation software capable of producing unstructured meshes for numerically efficient and robust calculation was needed. The numerical mesh generation software must be able to adapt the numerical mesh to a relatively large number of geological, hydrological, and topographical features of the system that are simulated, which can be hydrofacies outlines, geological faults, water body outlines, steep slopes, areas of different land cover and land use, as well as water use infrastructure such as pumps and drains. This approach ensured that important hydrological features were well represented while minimizing the total number of computational nodes.

In this study, we used the commercial mesh generator AlgoMesh (HydroAlgorithmics Pty Ltd; version 2.0.20.32621; https://www.hydroalgorithmics.com/software/algomesh), which generates high-quality grids for multiple finite-volume and finite-element simulation software. Alternatively, the open-source 3D finite element grid generator Gmsh^94^^,^^95^ (http://gmsh.info) could be used.

#### Integrated surface-subsurface hydrological modeling software

An Integrated Surface-Subsurface Hydrological Modeling (ISSHM) software was required for simulating the full water cycle—capturing interactions between rainfall, surface runoff, infiltration, and groundwater flow—to support accurate water resource management and impact assessments under changing climate conditions. One of ISSHM’s core strengths is their ability to explicitly simulate the interactions between surface water and groundwater with spatially heterogeneous parameters, which is critical for adequately representing hydrological processes in complex geological settings such as alpine catchments and volcanic islands^6,96^. Another key advantage of ISSHMs is their ability to explicitly simulate solute transport throughout the surface and subsurface, as well as across their interface, which enables advanced model calibration and deeper interpretation of environmental tracer data across multiple sources^10,97–105^. These capabilities make ISSHMs particularly valuable for predictive modeling, especially when evaluating the effects of climate change on groundwater systems^106–108^. However, it should be noted that the current model resolution, designed to balance geological detail with computational feasibility, may limit its immediate suitability for explicit solute transport simulations. Users aiming to apply the model for such purposes should consider refining the mesh or adapting the model structure accordingly. Alternatively, simplified transport analyses can be readily achieved with the current model structure using a particle tracking approach, which relies exclusively on the advective flow field^109^.

In this study, we used the research-oriented commercial software HydroGeoSphere (HGS) (Aquanty Inc.; revision 2817; https://www.aquanty.com). In HGS, surface and subsurface flow as well as heat and mass transport are simulated in a fully integrated manner under consideration of 2D surface flow, variably saturated subsurface flow, discrete fracture flow, vegetation-land-water interactions, density-dependent flow and transport, as well as winter hydrological processes - all processes which are relevant for the studied site^97,98,100,110–112^. Alternatively, the open-source software ParFlow^113,114^ (https://parflow.org) could be used.

#### Computational fluid dynamics, visualization and processing software

A visualization and processing software was required to interpret, analyze, present and communicate the results of hydrological simulations in a clear and accessible way. These tools allow users to process large and complex datasets, create detailed plots and extract meaningful patterns from spatial and temporal model outputs.

Here, Tecplot 360 (Tecplot, Inc.; version: 2024.R1; https://tecplot.com), a powerful commercial software for 3D visualization, and post-processing, of large numerical models was used. Tecplot is the recommended visualization and postprocessing tool for HGS, enabling efficient exploration of simulation results, including groundwater flow paths, concentration fields, and time-series data. Alternatively, the open-source, multi-platform data analysis and visualization application ParaView (https://www.paraview.org) can be used.

### Workflow

**Fig. 2:**
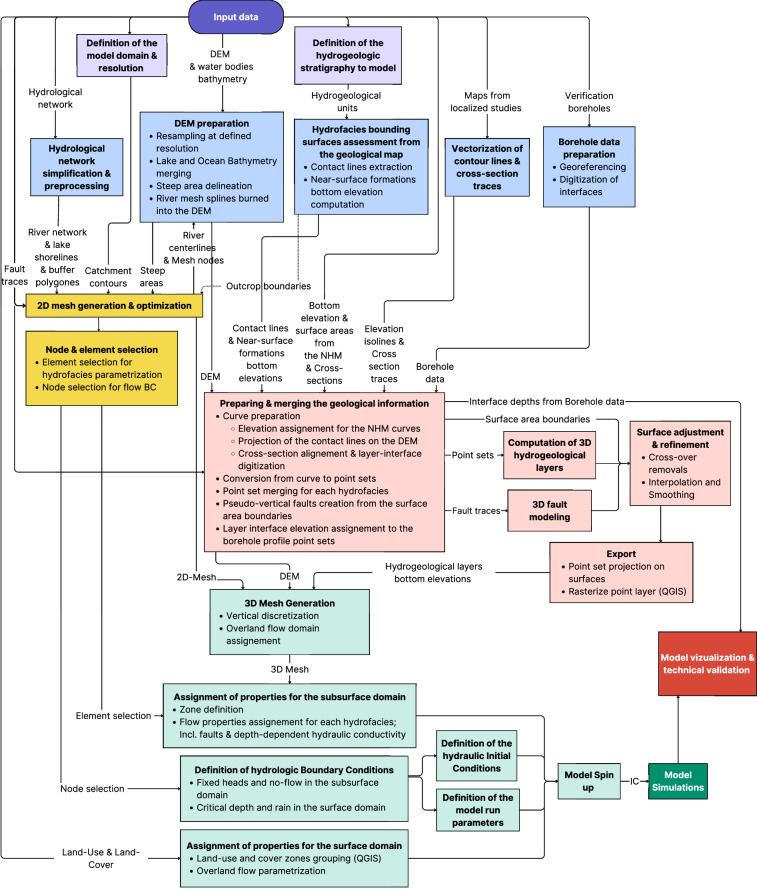
Workflow for developing an integrated surface–subsurface hydrological model in geologically complex volcanic watersheds. Each colored box corresponds to a major process described in the main text: conceptualization (purple), input data processing (blue), 3D geological modeling (pink), numerical mesh generation (yellow), 3D integrated hydrological modeling (green). Data transferred between steps is indicated along the connecting arrows.

Figure 2 illustrates the workflow for developing a 3D geological model and a 3D integrated surface-subsurface hydrological model in geologically complex volcanic watersheds. The process, which is presented in detail in the sections below, started with the collection, organization, and preprocessing of all available geological and hydrofacies data using a GIS software. In parallel to the input data pre-processing, a numerical mesh was generated and optimized using a numerical mesh generation software. In the next step, this data was imported into a 3D geological modeling software, where stratigraphic units, faults, and hydrofacies boundaries were interpreted and assembled into a geologically coherent 3D model. Modeled hydrofacies boundaries were then exported back to the GIS software for rasterization. Finally, the rasterized hydrofacies, numerical mesh, and hydraulic input data (such as flow boundary conditions and parameters) were integrated into the hydrological model. Flow properties, as well as initial and boundary conditions were assigned to the subsurface and surface domains, and the simulations were controlled by a set of running parameters to produce a catchment-scale integrated surface-subsurface hydrological model of Mt. Fuji watershed. The resulting outputs were then visualized and reviewed for validation by comparing them with observations.

### Conceptualization

#### Definition of the model domain and resolution

The surface model domain, representing the Mt. Fuji catchment, was delineated to represent the topographic catchment using the “*catchment*” tool in QGIS. The resulting catchment area spans approximately 1,650 km². Vertically, the model extends from 3,776 m asl, corresponding to the summit of Mt. Fuji, to −1,100 m asl, representing the bottom of the Neogene Fujikawa, Nishiyatsushiro, and Tanzawa groups, that is, the bottom of Mt Fuji’s basement (BS). The model was vertically cut at the shoreline of the Suruga Bay.

The optimal model resolution depends heavily on the resolution, distribution and quality of the base data sets. In the case of Mt. Fuji, the data sets (i.e. geological map and NHM) used to create the hydrogeological framework form the upper limit for the model resolution. For example, the geological map of Mt. Fuji^59^ with a resolution of 1:50,000 shows geological details of 50 m in diameter at this map scale, with a size of 1 mm. A finer resolution, therefore, would not add geological accuracy and could lead to misleading model detail.

From a numerical stability perspective, there is a trade-off between a fine mesh that may create unfeasibly long runtimes due to the many discrete points to calculate, and a coarse mesh, which may result in numerical instability due to the resolution being too low to resolve the physical behavior that is simulated. Considering the large spatial extent of the model domain, a horizontal resolution of 75 m was selected to strike a balance between sufficient spatial detail, numerical stability, and acceptable computational performance (i.e., simulations finishing within hours to days).

#### Definition of the hydrogeologic stratigraphy to be modeled

The hydrogeological stratigraphy of Mt. Fuji catchment is based on the hydrofacies classification presented in Table 1.

### Input data processing

In the geological modeling software used in this study, the base elevation of each hydrofacies must be imported as a vector dataset (.shp) to allow construction of a 3D model. As a result, all spatial datasets must be preprocessed and formatted within a GIS software before import. A critical step in this process is ensuring that all geospatial data share a consistent coordinate reference system to maintain spatial accuracy during model construction.

#### Hydrological network simplification and preprocessing

The base hydrological network dataset includes all rivers, streams, and detailed lake shorelines. However, incorporating the full-resolution dataset into the numerical mesh would result in excessive refinement and prohibitively high computational costs. To address this, the data were preprocessed in QGIS. Only the main river segments (stream orders 1 and 2) were retained, and both river and lake shoreline geometries were simplified using the “Simplify” tool to reduce vertex density while preserving essential hydrological features. These simplified line features were then exported to the meshing software and used to guide local mesh refinement. Additionally, buffer polygons were generated 100 m around the selected river segments and lake shorelines to further control the mesh element size during mesh optimization.

#### DEM preparation

The surface topography and bathymetry of lakes and coastal areas define the top boundary of the model. As in the raw DEM, elevation values over water bodies correspond to the free surface water level; the Fuji Five Lakes were merged with the DEM to accurately represent submerged terrain. To match the DEM to the model resolution, the merged 30 m DEM raster was upscaled to a horizontal resolution of 75 m using the “SAGA resampling” tool in QGIS, applying mean-value aggregation.

As necessary for all physically based surface water flow modeling, the DEM had to be further refined in parallel to the mesh generation in order to ensure consistency between DEM coarsening and mesh refinement^115^. Specifically, river centerlines generated in the 2D mesh had to be reconsidered in the DEM to prevent artificial blocking of surface water flow along the stream network, which would be caused by DEM simplification linked to the mesh’s coarseness at this spatial scale. This was achieved by stream-burning using the r.carve tool from the GRASS toolbox in QGIS. Parameters included a 100 m channel width, a 2 m channel depth, and the exclusion of flat flow paths to preserve hydrological connectivity.

To further support mesh refinement in terrain where significant hydraulic gradients are expected, a set of polygons encompassing all areas with slopes greater than 20% was generated.

Finally, a regular point grid (.shp) with 75 m spacing was created in QGIS, centered on raster pixels, and used to sample elevation values from the DEM. This point dataset was then exported to Aspen SKUA for geological modeling.

#### Hydrofacies bounding surfaces assessment from the geological map

The geological map of Mt. Fuji provides polygon vector data of the outcropping geology across the entire catchment. To incorporate this information into ISSHM modeling, the original map—comprising numerous geological units—was first simplified by grouping formations into relevant hydrofacies (Fig. 1c) and merging them into a single polygon shapefile for each hydrological unit. To estimate the base elevation of each hydrofacies, the following principle was applied: where a formation is in contact with an older unit, the surface trace of the boundary between the formations represents the base of the younger formation. Therefore, the relevant contact lines were extracted as vector shapefiles and exported to the geological 3D modeling software.

Additionally, the surficial hydrofacies AL and MF were largely absent from the coarse NHM despite their dominance in the outcropping geology across much of the catchment. In agreement with the information available from the NHM and with localized modeling studies^31,116^, a linearly increasing thickness from 0 m at the summit of the volcano to 10 m for AL and 20 m for MF at a radial distance of 12 km from the summit, i.e., the center of the plains around Mt. Fuji, was assumed. To incorporate this assumption into the 3D geological model, a regularly spaced point grid was generated in QGIS, and surface elevation values were extracted from the DEM. A spatial linear function was then applied at each point to estimate the bottom elevations of AL and MF based on terrain elevation. The resulting dataset was clipped to include only areas where AL and MF were present according to the geological map, assuming that MF underlies AL wherever AL is present.

### Vectorization of the contour lines and cross-sections

Some local studies provided information about the bottom elevation of hydrofacies in the form of contour maps or (hydro)geological cross-sections (Table 2). To use this information in the 3D geological modeling, the contour lines and cross-sections needed to be vectorized in QGIS. First, the maps are georeferenced to the chosen Coordinate Reference System (CRS). The isolines of the bottom elevation of each hydrofacies are then manually captured in a vector file, with the elevation of each line reported as an attribute. For the cross-sections, the traces are manually captured as lines in a vector file.

### Borehole data preparation for the model verification: georeferencing and digitization of layer interfaces

For geological model verification, borehole data from some geographically localized studies were used (Table 2). For this purpose, borehole locations must be compiled into a point vector layer. Subsequently, the borehole logs are interpreted to identify the depth of each of the hydrofacies present in a log, and these points are then added as an attribute to each respective borehole point in the vector layer.

### 3D geological modeling

All processes in the geological modeling software Aspen SKUA, including data import and processing, are performed manually to enable greater control and customization during model construction.

#### Importing the data

Point and line vector layers were imported into Aspen SKUA as “cultural data”. To maintain organization within the project, these inputs were grouped into feature categories corresponding to each hydrofacies.

The DEM and borehole location point sets, isolines representing the surface bottom elevations and areas from the NHM, and fault traces were imported directly from vector files. Cross-section images, trimmed to their geological boundaries, were imported as 2D voxets. Additional vector line data — including contact lines and bottom elevation of near-surface formations from the geological map, isolines from localized geological studies, and cross-section traces prepared in QGIS — were also imported for further processing. Note that in Aspen SKUA, imported line vector layers are referred to as “curve sets”.

#### Preparing and merging the geological information

For the isolines from the NHM, the script editor tool in Aspen SKUA was used to assign elevation values as curve properties. The contact lines from the geological map were vertically projected onto the DEM. For the cross-sections, the imported voxets were resized using control points by selecting the trace edges and manually adjusting their elevation based on the DEM and the depth indicated in the cross-section. Once correctly aligned, a new 3D curve was digitized by placing segments along the image to trace the bottom elevation of each hydrofacies.

All resulting curves were then converted into point sets. For each hydrofacies, a single merged point set was created by combining all relevant inputs. The only exception is for layers displaced by the FKFZ faults — specifically BS, OLF, and OLFM — for which two separate point sets were created to represent the east and west sides of the fault zone.

The curves defining the surface area boundaries from the NHM were used to generate pseudo-vertical faults. These extended well beyond the vertical limits of the model domain and were used to clip the hydrofacies surfaces in a later step.

Finally, a separate point set was created for each of the MF, FV, and OLFM layers, whose interfaces were also recorded in borehole profiles. As with the isolines, elevation values were assigned using the script editor tool based on the attribute tables of the imported points. These data were used solely for technical validation of the model and were not included in the actual model computations.

#### Computation of 3D hydrofacies layers

The bottom surfaces of the hydrofacies layers were computed explicitly through direct triangulation of these point sets. This method enables manual adjustments based on geological interpretation, which is especially valuable in geologically complex regions. Unlike implicit modeling, which relies on algorithm-driven interpolation and includes statistical uncertainty estimation, this explicit approach offers greater transparency and control.

#### 3D fault modeling

Fault traces were used to construct vertical fault surfaces in Aspen SKUA using the software’s dedicated fault modeling tool. Each fault was represented as a single-element vertical surface extending continuously through all geological layers, ensuring structural consistency across the model.

### Surface adjustment and refinement

After computing the geological surfaces, crossing surfaces were corrected in accordance with the stratigraphic sequence, where more recent geological layers overrule older layers. A key constraint in the modeling process is that no layer can exist above the outcrop of its preceding units. To enforce this, the surfaces were trimmed using the outcropping extents of the lower layers—implemented in Aspen SKUA by cutting one surface with another.

The surface area boundaries from the NHM were treated as minimum extents, allowing for the possibility that layers, where warranted by the other available data, extended beyond them. The final horizontal extent of each layer was determined based on the distribution of outcropping formations.

**Fig. 3:**
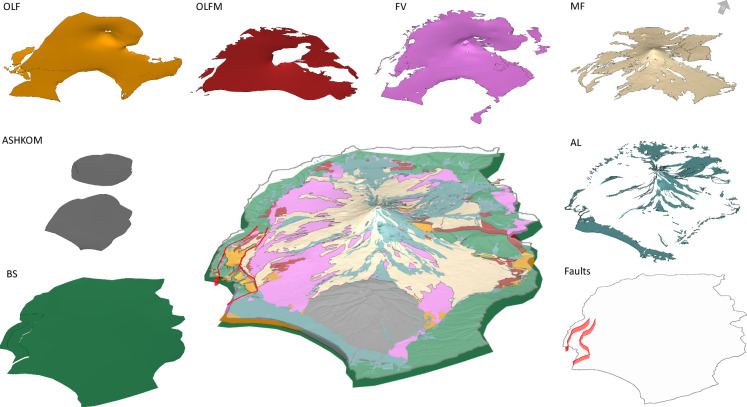
3D geological model snapshot, taken in Aspen SKUA, with a factor two of vertical exaggeration. The layers are the bottom elevation of each hydrofacies: BS (forest green), ASHKOM (grey), OLF (orange), OLFM (dark red), FV (pink), MF (gold), AL (blue). The fault traces and surfaces are depicted in red.

Respecting the mapped outcrops and contact lines proved especially challenging in the fault zone, where layers had been displaced or eroded. Manual adjustments were made to maintain the correct stratigraphic relationships while honoring mapped surface geology. Where needed, interpolation was used to smooth out numerical artifacts. Finally, surface smoothing and decimation were applied to simplify layer boundaries, using a vertical tolerance of 1 meter. Figure 3 presents the final geological model constructed in Aspen SKUA.

#### Export

Once a 3D geological model is finalized in Aspen SKUA, it needs to be exported in a format compatible with the selected hydrological simulation software. Here, we used the finite element ISSHM HGS, which requires hydrogeological layer interfaces (i.e., hydrofacies surfaces) to be provided as raster files. A key consideration during export is ensuring sufficient spatial resolution to accurately represent complex geological features, such as fault zones. To satisfy this requirement, we used a regular point grid with a horizontal resolution of 75 meters—the same grid originally created for exporting the DEM from QGIS to Aspen SKUA. Elevation values for each layer were assigned using a vertical projection method, starting from the DEM and progressing downward through the stratigraphy. In areas where a geological unit was absent, its volume was artificially “closed” by the bottom of the overlying hydrofacies, ensuring that each layer spans the entire horizontal model extent, as required by finite element models like HGS. The processed point grid was then re-exported to QGIS, where it was interpolated into raster format (TIN interpolation tool with 100 m resolution) for input into the HGS modeling workflow.

### Numerical mesh generation

To construct a comprehensive 3D model of the entire catchment within a hydrological simulator, the first step is to generate a 2D numerical mesh. This mesh discretizes the horizontal extent of the simulation domain at a resolution that balances spatial accuracy and computational efficiency while incorporating constraints from geology, hydrology, topography, land use/cover, and water infrastructure. In the second step, a 3D mesh is built by expanding the 2D mesh vertically using the interface elevations of the various hydrofacies layers.

### Horizontal mesh generation and optimization

Although HGS supports both finite element and finite difference methods, the present model was developed using the finite element approach. Accordingly, mesh generation was carried out with the finite element mesh generation software AlgoMesh using line and polygon vector files as input. The catchment contours, resampled at a 400 m resolution, define the outer extent of the mesh. Simplified river centerlines, lake shores, and fault traces were also resampled at 400 m resolution and incorporated into the mesh.

To guide mesh refinement during optimization, steep-slope zones and buffer polygons around the hydrological network were used to apply spatially variable constraints on maximum edge length. These were set to 200 m near rivers and fault traces, 250 m along lake shores, and 500 m in steep-slope areas. The maximum edge length across the rest of the domain was set to 750 m.

The final refined 2D finite element mesh, which contains 24,800 nodes and 49,044 triangular elements, with nodal spacing ranging from 185 to 750 m, was then exported in the AlgoMesh-HGS interchange format A2H, which is based on standard ASCII mesh file structures. River centerlines and mesh nodes were also exported as vector data for subsequent use in DEM preprocessing.

While ideally, the mesh would also be refined using the outcropping boundaries of geological layers to better align surface elements with mapped formations, this was omitted here to avoid excessive refinement, given the complexity of the geological map. Doing so would have significantly increased computational costs.

### Node and element selection

During the ISSHM model setup, initial parameter values representing the system’s physical properties were assigned to nodes and elements of the finite element mesh. Therefore, geological and hydrological features were spatially mapped to the mesh in AlgoMesh, utilizing line and polygon shapes generated in QGIS, which enables the targeted selection of relevant nodes and elements.

Flow boundary conditions were applied to specific node sets located along the model boundaries, notably along the Suruga Bay coastline, the catchment perimeter, and river outflow points. Hydrofacies were parameterized by using the polygon element selections and expanding these in the vertical dimension to enclose all numerical model layers over which the respective hydrofacies were assumed to exist. Elements adjacent to the fault lines were also selected for the hydrogeological parametrization.

All node and element selections were exported from AlgoMesh in HGS-compatible formats (.nchos for nodes and.echos for elements).

### Integrated hydrological modeling

#### 3D mesh generation

In order to preserve fine-scale surface features such as riverbeds and prevent the formation of artificial depressions or sinks, the elevation of the surface layer of the model was defined by directly mapping the elevation values from the fully processed DEM (which includes lake and coastal bathymetry as well as burned streams) at each given node location to that respective node by importing the 2D finite element node locations and using the raster value extraction tool in QGIS.

Elevations of the hydrofacies interfaces were defined by first generating layers for each hydrofacies bottom in HGS using the “generate layers interactive” sets of commands and by subsequently using the “elevation from raster” command. The mapping was done bottom to top, whereby the model base was defined first using the bottom elevation of the BS hydrofacies. Moving upward in the lithological sequence, the bottom surface of the overlying hydrofacies was defined according to the same principle (thereby making this layer also the top of the underlying hydrofacies). Due to the discontinuous nature of some hydrofacies, overlapping elevation values occasionally occurred between interfacing hydrofacies. In such cases, a minimum enforced thickness of 5 cm was applied to maintain stratigraphic structure.

The near-surface layers are subject to more dynamic flow processes, such as infiltration through unsaturated zones and rapid water table fluctuations. As a result, they require finer vertical discretization than deeper layers^96^. To capture this variability, each hydrofacies layer was subdivided into 5 to 7 evenly spaced sublayers, with sublayer thicknesses varying according to the total vertical extent of the hydrofacies at each location. This resulted in a total of 40 finite element layers across the model. In the ISSHM HGS, overland flow is simulated explicitly on top of the model surface, that is, on the topmost layer.

#### Assignment of hydraulic properties for the subsurface domain

**Fig. 4:**
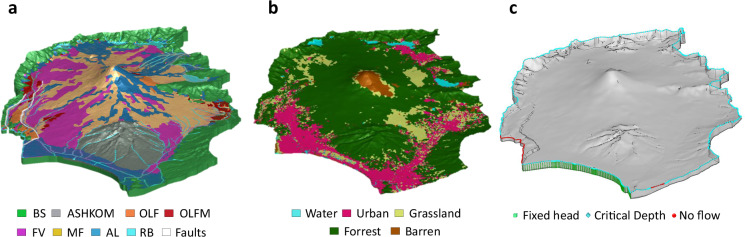
Set up for the integrated hydrogeological model of Mt. Fuji catchment (Japan); (**a**) Elemental zones in the porous medium domain, corresponding to the different hydrofacies in Table 1, as well as the lake and riverbeds (RB) and fault elements, corresponding to the lake and river beds, resp. to the fault zone; (**b**) Elemental zones for the overland flow domain, corresponding to the categories in Table 3; (**c**) Model boundary conditions: blue diamonds represent the critical depth boundary conditions for the surface domain in the Suruga Bay area, on the river outflows and on the catchment contours. The Fuji River Termination (SW) and Kise River effluent (SE) are set to no-flow boundary conditions (red spheres). The green boxes represent the fixed head for the subsurface domain in the Suruga bay area, and to the river elevation in the North and East outlets (not visible from this point of view). For the lateral subsurface boundaries for which no other boundary conditions are indicated, as well as for the base of the model, no flow conditions were imposed (not shown).

Assigning material properties in HGS requires defining zones that group all elements associated with a specific hydrofacies in the porous medium. This was achieved using spatial queries within HGS. The vertical extent of each zone was defined by the corresponding mesh sublayers established during the 3D mesh construction phase. The horizontal (plan-view) extent was determined by selecting elements in AlgoMesh. Starting from the base of the model, zone numbers were assigned to each hydrofacies sequentially. The final zone configuration is shown in Fig. 4.

The hydraulic properties assigned to each hydrofacies are summarized in Table 1, with the exception of the hydraulic conductivity of BS and ASHKOM. As part of the sensitivity analyses (described hereafter), depth-dependent hydraulic conductivity was implemented for these hydrofacies following the approach proposed in the literature^117^, which characterizes permeability as an exponentially decreasing function with depth. This method is particularly suited for volcanic basement formations, where high near-surface permeability due to fracturing decreases due to increasing overburden stress and mineralization. Given the similarity of the BS and ASHKOM layers to the volcanic units of the Oregon Cascades, the following equation was applied for depths z < 1.5 km:

1 $$K\left(z\right)={K}_{s}\cdot {e}^{-z/d}$$

Where *K(z)* [m d^−1^] is the hydraulic conductivity at depth *z* [m], *K*_*s*_ is the near-surface hydraulic conductivity [m d^−1^], and *d* is the characteristic depth of permeability decrease [m]. Using the values *K*_*s*_ = 4.3 m d^−1^ and d = 75 m, this formulation ensures a realistic permeability decrease with depth while maintaining a finite value at the surface. At depths greater than 2,000 m, the calculated values from this approach converge with those reported in Table 1, ensuring consistency with previously established parameterizations for deep basement formations. The chosen parameters are consistent with observed permeability structures in volcanic basement rocks^117^. In the model, this was implemented by first calculating the coordinates of each element’s centroid, determining its depth, and then applying Eq. 1.

In addition to the hydrofacies described in Table 1, to better represent surface–subsurface interactions, the elements beneath lakes and rivers (i.e., lakebed and riverbed sediments, referred to as RB in Fig. 4) in the uppermost model layer were assigned a reduced hydraulic permeability (K = 10^−8 ^m s^−1^). This reflects the presence of fine-grained, low-permeability lacustrine sediments as observed in Lake Yamanaka and Lake Motosu^118,119^ and addresses the fact that riverbed sediments generally exhibit a lower conductivity than the surrounding alluvial sediments due to deposition of fines and the subsequent formation of a colmation layer^102,120^.

#### Assignment of hydraulic properties for the fault zone

In volcanic watershed models, faults can significantly disrupt the continuity of hydrofacies layers. Depending on their composition and structural characteristics, faults may act either as barriers (e.g., due to low-permeability fault gouge or clay-rich lithologies) or as preferential flow conduits. Stratovolcanoes are typically located in tectonic constellations such as active subduction zones. In such locations, incorporating 3D fault structures is essential for preserving the geological integrity of hydrofacies distributions and ensuring the reliability of surface–subsurface hydrological simulations.

At Mt. Fuji, the influence of the Iriyamase segment within the FKFZ on groundwater dynamics—specifically on total hydraulic head distribution and salinity—has been investigated using conceptual and numerical models^70^, which indicate that this fault zone exhibits high permeability. Based on these findings, elements adjacent to the modeled fault traces, extending through the entire vertical profile, were assigned elevated hydraulic conductivity values (K = 10^−3 ^m s^−1^; see Fig. 4).

While HGS supports discrete fracture network (DFN) representations using dual- or multiple-permeability domain^121^, an implicit fault-zone parameterization was chosen for computational efficiency. This choice prioritizes scalability and model performance while still honouring the key hydrogeological role of major fault structures.

#### Sensitivity analysis of subsurface hydraulic properties

Initial simulations with spatially uniform K values and no sediment layers resulted in widespread, unrealistic water retention patterns, including widespread surface ponding in topographically rugged areas and overly persistent saturation. Subsequent incorporation of depth-dependent K values^117^ improved subsurface flow behavior but caused simulated lakes to drain fully. To address this, lakebed sediment information^118,119^ was incorporated, introducing a low-permeability layer that restored realistic lake representation. Finally, reduced-permeability riverbed sediments were included to improve surface-subsurface dynamics^102,120,122^. These iterative refinements improved model realism and numerical stability, highlighting the importance of depth-dependent K values and surface waterbed resistance in establishing a robust base configuration for future use and calibration.

#### Assignment of hydraulic properties for the surface domain

The surface domain is parametrized using the LULC dataset. The original LULC dataset considered 14 distinct land-use types, which for the purpose of this ISSHM modeling exercise were aggregated into five broader categories based on their influence on surface hydrological processes such as infiltration, runoff, and evapotranspiration, using QGIS.

The first category, Water and Wetlands, includes water bodies and wetlands. Impervious Urban Areas consist of built-up areas, solar panel installations, and greenhouses, and are characterized by low infiltration rates and high surface runoff. The Grassland and Agriculture category encompasses paddy fields, croplands, and grasslands, which exhibit moderate infiltration. Forests, including deciduous broad-leaved, deciduous needle-leaved, evergreen broad-leaved, evergreen needle-leaved, and bamboo forests, have high infiltration capacities and are expected to play a significant role in canopy interception and evapotranspiration. Lastly, the Barren Areas category, represented by bare ground, is characterized by low infiltration and high runoff potential. The parameters for each zone are summarized in Table 3. In HGS, the zones were implemented by reading the dominant class directly from the aggregated raster file and are shown in Fig. 4b.

**Table 3:** Surface flow domain properties for the different land-use categories.Manning’s roughness coefficients are based on established values from the literature (refs. ^130,131^).

Land-use	Manning’s roughness coefficient [m^−1/3^ s]	Rill storage height [m]	Obstruction storage height [m]
*Water and Wetlands*	0.1	0.002	0.0
*Urban areas*	0.02	0.05	0.005
*Grassland and Agriculture*	0.1	0.01	0.05
*Forest*	0.04	0.05	0.10
*Barren areas*	0.02	0.002	0.015

Rill and obstruction heights were informed by values from ref. ^126^ for similar terrain and adjusted through model-based trial-and-error to achieve hydrologically realistic surface flow behavior in the Mt. Fuji catchment.

Surface–subsurface hydrological interactions in HGS are defined to be computed using the dual node approach^121^. A uniform exchange length *l*_*exch*_ [m] coefficient of 0.1 m was considered for the entire model domain, striking a balance between ensuring smooth exchange fluxes between the surface and subsurface domains and guaranteeing numerical stability.

#### Definition of hydrologic boundary conditions

For the subsurface domain, boundary conditions (BCs) were assigned as follows: Dirichlet boundary conditions (constant head) corresponding to 710 m asl and 260 m asl were defined along the lateral model boundaries of the Katsura and Ayuzawa river valleys located in the North and East of the catchment. In the South all along the subsurface interface to Suruga Bay, a constant head of 0 m asl (freshwater equivalent) was employed, allowing for both submarine groundwater discharge and drainage through the Fuji and Kano rivers. A freshwater head was used instead of a seawater-equivalent head because the system is SGD-dominated^56^, salinity effects are negligible on the scale of the hydraulic gradients, and density-driven flow was not simulated. No-flow boundaries were assigned to the remaining lateral and bottom subsurface boundaries of the subsurface domain (Fig. 4c) as the model domain is based on the topographic catchment, and no data are available to justify deeper or transboundary flows.

For the surface domain, a critical-depth boundary condition was applied along all boundaries, allowing water to exit the model freely. Two specific sections of the lateral model boundary require distinct treatments:

1. Fuji River Termination (SW Border) – The Fuji River, originating within the catchment, follows the catchment’s southwestern edge before discharging into Suruga Bay. It is fed by both the Fuji catchment and contributions from the mountain ranges on the western side
2. Kise River Effluent (SE Border) – A short section of the Kise River briefly exits the catchment before re-entering. Before re-entering, it is joined by the Kano River, which originates in the Izu Peninsula.

By treating these sections as no-flow boundaries, all the water contributed to these rivers by Mt. Fuji catchment could be retained within the model domain until the rivers reached their proper outlets. As this underestimates the total discharge of these river systems at their outlets, river discharge observations for the Fuji and Kano rivers into the Suruga Bay area cannot be directly used for model parametrization. This treatment also pragmatically accounts for the FKFZ along the southwestern boundary, which connects to the Fuji River catchment but lies outside the scope of this model.

Evapotranspiration was not explicitly simulated, both to reduce computational complexity and because the primary focus of this study is on structural model development, rather than detailed surface energy or water balance processes. Actual evapotranspiration, which amounts to approximately 1000 mm yr^−^¹ (ref. ^47^), was, however, considered by subtracting it from the total average of 2500 mm yr^−^¹ of precipitation that falls on Mt. Fuji catchment each year^50^ and by applying a uniform rainfall rate of 1600 mm yr^−^¹ throughout the entire catchment.

#### Definition of hydrologic initial conditions, model spin-up and model run parameters

For the initial model spin-up, contour lines of the mean hydraulic heads in Mt. Fuji catchment available through the Water Environment Map of Mt. Fuji platform^123^ are digitized in QGIS, rasterized, and then used as input to define the initial head distribution in the subsurface domain in HGS. For the surface domain, an initial water depth of 0.00 m is used (i.e., default parametrization). Once the hydraulic heads have reached the quasi-steady state (approx. 10,000 yrs of simulation), the resulting hydraulic head distributions in the subsurface and on the surface are iteratively used as initial conditions for subsequent simulations with more refined parameterizations.

Simulations are controlled using a Newton-Raphson solver and an adaptive time-stepping scheme, starting from an initial time step of approximately 1 microsecond with a maximum timestep of 100 days for a total simulation duration of 3,000 yrs. This is sufficiently long to reach a new quasi-steady-state in all hydrogeologic formations for simulations started using the quasi-steady state heads of the spin-up model. The Newton absolute and Newton residual convergence criteria of HGS are set to 10^−2 ^m.

Using this setup, a simulation of Mt. Fuji catchment of 3,000 yrs with constant boundary conditions takes about 24 h to reach a new quasi-steady-state using 6 CPU in parallel on a regular desktop with an Intel i9-12900K processor and 32 Gb of DDR5 RAM. Export of model results

The 3D ISSHM model geometries, numerical mesh, hydraulic parameters, boundary conditions, and model outputs were exported from HGS in Tecplot and Paraview-compatible format for post-processing, visualization, and technical validation. These files are available in the associated repository^91^.

## Data Records

All model data generated in this study are openly available from a HydroShare repository 10.4211/hs.ac6f9e551d934bd9b5706102f9a4cb5d and structured into two primary categories: (1) the input files not otherwise publicly available, (2) the 3D geological model, and (3) the 3D ISSHM model^91^. Original input datasets publicly accessible from external sources are described in the Methods section and referenced by URL in Table 2.

## Model Files

The geological model is exported as a series of raster files, each representing the base elevation of individual hydrofacies as defined in Table 1. These files can be used directly in hydrological modeling software such as HydroGeoSphere. In addition, the native Aspen SKUA project files (i.e., DXF) are provided for users who wish to inspect or modify the geological model in a geological modeling software environment.

The integrated hydrological model is archived as HydroGeoSphere-compatible input, run, and output files, including the 2D mesh, node and element selection, and raster files. The dataset allows users to rerun or adapt the model independently.

## Technical Validation

The accuracy of the 3D geological model was assessed by comparing modeled hydrofacies interfaces with independent borehole data. In addition, the 3D ISSHM, of which the 3D geological model forms the conceptual and geometrical foundation, was independently validated by comparing quasi-steady state simulated hydraulic heads with respective measurements.

## Geologic Verification

**Fig. 5:**
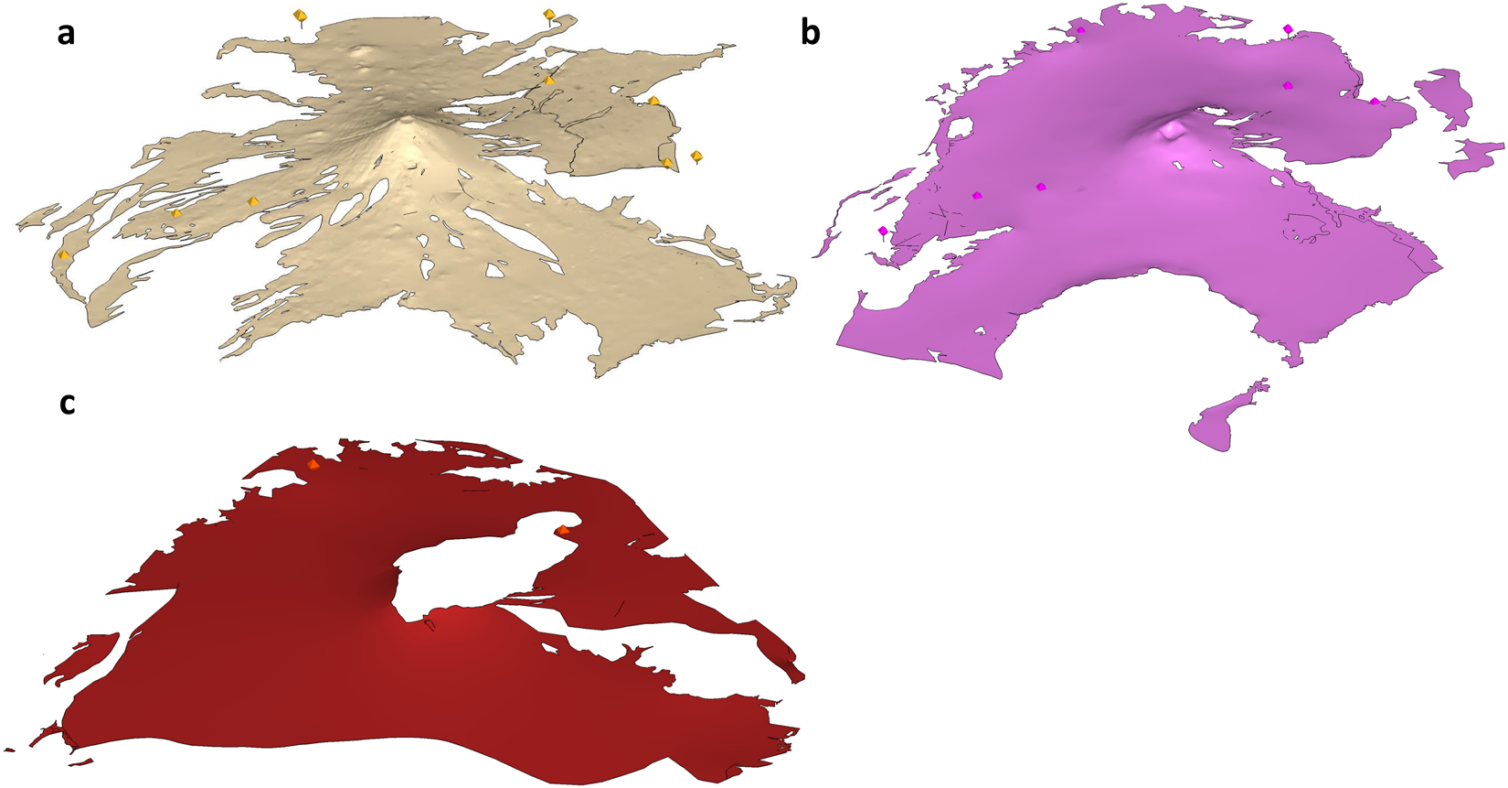
Visual comparison of modeled hydrofacies interfaces and observed layer depths from verification boreholes in Aspen SKUA. Colored surfaces represent hydrofacies boundaries, while overlaid diamonds indicate stratigraphic transitions recorded in the borehole logs for (**a**) MF, (**b**) FV and (**c**) OLFM. Boreholes which suggest the presence of a layer outside of the layer surface as developed in the geological model are indicated by the vertical lines.

A visual comparison was performed in Aspen SKUA between the computed layer interfaces and those recorded in borehole profiles. Figure 5 illustrates snapshots from the model, overlaid with borehole points marking the recorded depths of key hydrofacies interfaces. Overall, the correspondence is satisfactory, especially given the catchment-scale resolution and the region’s geological complexity.

However, this verification is limited by several factors. The geology of the Mt. Fuji catchment is highly complex, with multiple superposed basaltic layers that are often lithologically similar, making it difficult to interpret borehole logs definitively. In many cases, assumptions were required to attribute specific depth intervals to modeled hydrofacies. Furthermore, the number of available verification boreholes was severely limited. Only a small subset of the findable borehole profiles is probing into sufficiently large depth to provide stratigraphic data for layers below the AL or MF. Despite these constraints, the comparison supports the structural reliability of the geological model and establishes a workflow that allows easy integration of additional verification data for future refinement. Ultimately, this also opens the possibility of introducing spatial heterogeneity into the hydrofacies parametrization, where data availability allows.

## Hydrologic Verification

The quality of the ISSHM is assessed by its ability to reproduce key hydrological indicators: the surface water network (i.e., river centerlines and lake characteristics) and groundwater hydraulic heads across the Mt. Fuji catchment. To this end, we compared a quasi steady-state simulation of 3,000 years of constant forcings derived from the hydrometeorological present-day mean with hydrological network data from the Ministry of Land, Infrastructure, Transport and Tourism^87,88^, and mean groundwater heads from the Water Environment Map of Mt. Fuji^123^.

**Fig. 6:**
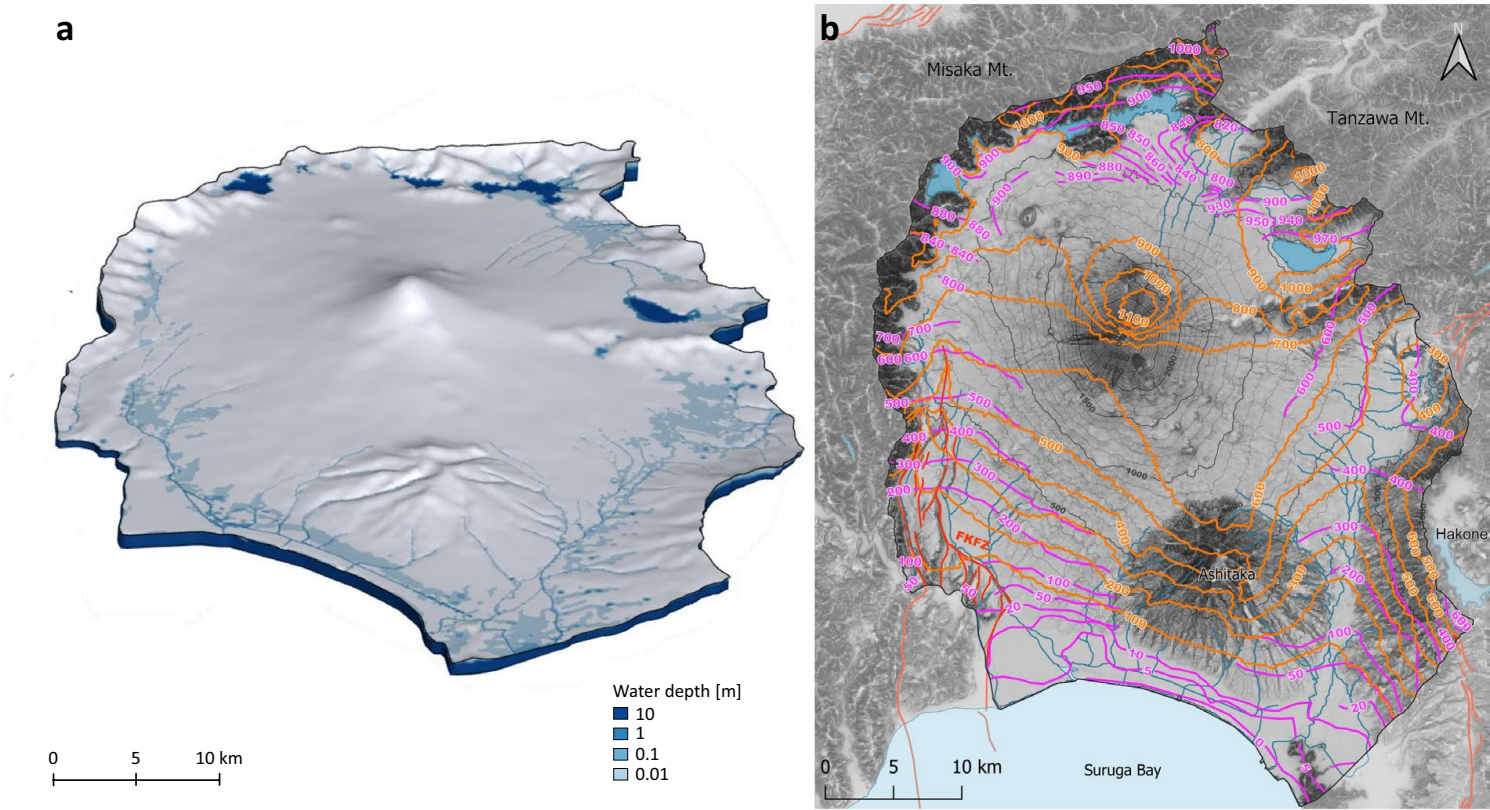
(**a**) Simulation output for water depth in the surface domain, and the saturation in the subsurface domain; (**b**) Comparison between observed (pink) and simulated (orange) hydraulic heads in Mt. Fuji catchment. FKFZ denotes the Fuji-Kawa Fault Zone.

Figure 6a shows simulated surface water depths and subsurface saturation. The model correctly reproduces the extent of the Fuji Five Lakes, with simulated maximum lake depths ranging from 130, 7, 73, and 30, 15 meters, west to east across the system (Lake Motosu, Lake Shoji, Lake Sai, Lake Kawaguchi, and Lake Yamanaka). These results are consistent with reported annual average depths of 120, 12.5, 70, 12, and 13 m^85^, indicating an acceptable level of accuracy.

Figure 6b compares simulated versus observed hydraulic heads. The model shows excellent agreement across the southern part of the catchment. In the northern and more mountainous areas, the pattern is more complex due to steep gradients and potential influences from surrounding ranges such as the Misaka and Tanzawa mountains. Nonetheless, the agreement remains satisfactory, particularly given the model scale and data constraints.

## Model Limitations

### 3D geological model

The geological model’s main limitation lies in the uncertainty about the robustness of the hydrofacies, resulting from the severely limited availability of deep borehole data. Unfortunately, due to the lack of data, this uncertainty is impossible to quantify. However, by building on data from the NHM, which represents a geologically consistent but national-scale (i.e., coarse) hydrological model, we incorporated a workflow that minimizes the uncertainty of the 3D geological model in the face of extreme data scarcity. The flexible workflow can accommodate a variety of geological data types, with their relative importance determined by the specific modeling context.

Another possible avenue to address the extreme data scarcity generally faced in volcanic systems would be through stochastic or rule-based 3D geological modeling approaches, such as those implemented in ArchPy^124^, or more generally in structural modeling frameworks that support uncertainty quantification^2,93^. While not applied in this study, such methods may be particularly suited to environments with sub-horizontal stratigraphy governed by well-defined depositional rules (e.g., Sedimentary Basins and, to some extent, Volcanic aquifers). Importantly, the positional uncertainty of hydrofacies interfaces may become less critical during parameterization, especially as flow calibration proceeds. These uncertainties ultimately propagate into hydrological predictions and can be evaluated through statistical calibration tools such as PEST^105,125^.

### 3D integrated hydrological model

The integrated hydrological model successfully captures major surface water features but also exhibits artifacts, such as artificial ponding in rugged terrain. These issues stem from mesh resolution limits tied to computational constraints. This highlights the importance of clearly defining the model’s purpose early on, allowing complexity to be adjusted accordingly. While trade-offs are inevitable, the model demonstrates that a well-structured, simplified setup can still yield realistic behaviour, often more effectively than an overly complex but poorly defined alternative.

At this stage, the model is not calibrated using automated multivariate inversion approaches and is therefore not suitable for predictive simulation^126,127^. Effective calibration would require spatially distributed observational data, which are often limited in volcanic terrain. However, the model was specifically designed to support automated calibration and scenario-based simulations.

Finally, although faults are structurally represented in the model, their effects on hydraulic conductivity and flow dynamics have not been analyzed. This remains an important topic for future investigation, with the current model as a robust starting point.

## Data Availability

The dataset is available in the HydroShare repository 10.4211/hs.ac6f9e551d934bd9b5706102f9a4cb5d^91^.

## Code availability

All input files required to run the reference model in the sensitivity analysis are available in the associated HydroShare repository^91^. To run the models, the HydroGeoSphere version from August 2022 (or onwards) and a valid license are necessary. A trial version of HydroGeoSphere can be obtained from aquanty.com
